# High pressure balloon dilation for vesicourethral anastomotic strictures after radical prostatectomy

**DOI:** 10.1186/s12894-015-0059-9

**Published:** 2015-07-02

**Authors:** Gen Ishii, Takehito Naruoka, Kanako Kasai, Kenichi Hata, Hiroshi Omono, Masayasu Suzuki, Takahiro Kimura, Shin Egawa

**Affiliations:** Atsugi City Hospital, 1-16-36 Mizuhiki, zip 243-8588 Atsugi City, Kanagawa Japan; Jikei University School of Medicine, 3-25-8 Nishishinbashi minato-ku, zip 105-8461 Tokyo, Japan

**Keywords:** Balloon dilation, Vesicourethral anastomotic stricture, Prostatectomy

## Abstract

**Background:**

Vesicourethral anastomotic stricture (VAS) is a rare but serious complication following radical prostatectomy (RP), and various types of managements for VAS have been proposed. We investigated the efficacy of transurethral balloon dilation in the management of VAS after RP.

**Methods:**

A total of 128 consecutive patients underwent open RP at our hospital between 2008 and 2013; of these, 10 patients (7.8 %) developed VAS. Transurethral balloon dilation was performed in all 10 patients, using a high pressure balloon catheter under fluoroscopic and endoscopic guidance. Follow-up endoscopy was performed, and patients in whom the stricture had recurred underwent repeat dilation. We retrospectively evaluated the management of VAS and short-term efficacy of high pressure balloon dilation.

**Results:**

The mean time from RP to diagnosis of VAS was 9 months (2–40 months); eight patients (80 %) were diagnosed within 6 months of RP. Balloon dilation of VAS was technically successful in all patients, and no perioperative complications were recorded. The median follow-up after balloon dilation was 24 months (7–67 months). There was no recurrence of VAS in eight patients (80 %) after the first balloon dilation, and all patients were controlled within the twice.

**Conclusion:**

High pressure balloon dilation is a highly effective and minimally invasive procedure for treating VAS.

## Background

Vesicourethral anastomotic stricture (VAS) is one of the most common complications after radical prostatectomy (RP), with rates ranging from 0.48-32 % [1, 2]. The development of a VAS can cause severe voiding dysfunction and result in significant deterioration of the patients’ quality of life.

Factors predisposed to the development of a VAS are not well understood, but they are reported to be related to prior transurethral resection of the prostate (TURP), the oncologic outcome, excessive blood loss, anastomotic ischemia, or urinary extravasation at the site of the VAS [3, 4]. Surgical techniques, including the suturing procedure and choice of bladder neck reconstruction, or bladder neck preservation, are also considered important factors [5].

There are various methods for managing a VAS, including simple or balloon dilation; cold knife incision, electrocautery incision or resection, and laser treatment. Among these options, cold knife incision is one of the most commonly used techniques, although optimal treatment remains controversial. While balloon dilation has also been used for managing VAS, previous studies have indicated that the outcomes of balloon dilation were poor, compared to those of alternative treatment modalities [6, 7]. We used the X Force® U30 balloon dilation catheter (Bard Medical Division), which can inflate up to 30 standard atmosphere (ATM). It provides sufficient dilation against even a severe VAS compared to conventional catheters. Therefore in this study, we assessed the efficacy of performing high pressure balloon dilation of VASs that occur in patients following RP.

## Methods

**Fig. 1:**
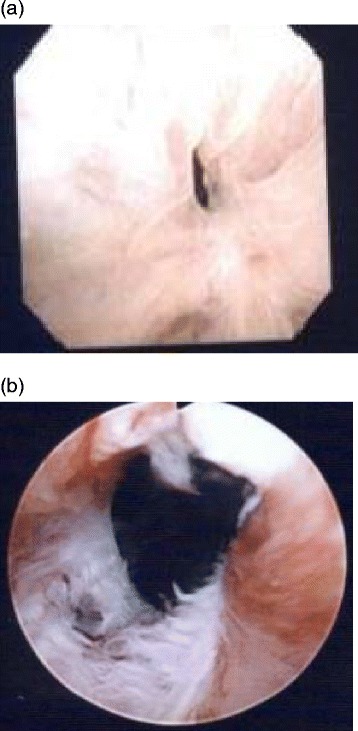
**a** a pinhole vesicourethral anastomotic stricture. **b** Endoscopic findings of the vesicourethral anastomotic stricture after successful balloon dilation

The study was approved by Atsugi City Hospital’s review board, and written informed consent was obtained from all patients. Data from 128 consecutive patients who had undergone open RP for clinically localized prostate cancer were enrolled between 2008 and 2013. Patients who complained of voiding difficulties (n = 10, 7.8 %) subsequently developed a VAS, and the presence of which was confirmed endoscopically in all cases (Fig. 1). All patients were treated with transurethral balloon dilation for VAS.

At the time of open RP, bladder reconstruction and eversion of the bladder mucosa had been performed as routine techniques, and vesicourethral anastomosis was performed using four interrupted sutures. Descriptive characteristics of the study population are shown in Table 1. In two cases, TURP was performed prior to RP. The median blood loss during RP was 1,387 mL, and five patients (50 %) had excessive bleeding (>1.5 L). Prolonged urinary leakage at the site of anastomosis occurred in 1 case. All the patients could void with a decent stream at the time of urethral catheter removal. None of the patients had suspected local recurrence of prostate cancer, but two who had extra prostatic extension (EPE) with resection margin (RM) received adjuvant radiotherapy for the prostate region.

**Table 1:** Demographic characters of patients with vesicourethral strictures (VAS) after radical prostatectomy (RP)

No. of Patient	10
Age (median)	70 (61–75)
Prior TURP	2
Gleason score	
6	3
7	6
unknown	1
EPE	2
RM	2
Blood loss at the time of RP (median)	1387 (775–3780)
Adjuvant radiotherapy	2

Transurethral balloon dilation was performed under regional anesthesia. We used a high pressure balloon catheter, the X Force®, which consists of a 6-French (Fr) open lumen, blunt-tip catheter, and 6-cm long balloon that inflates fully to 30 Fr at a maximum inflation pressure of 30 ATM. A 10-mL Eagle inflation device was also used.

**Fig. 2:**
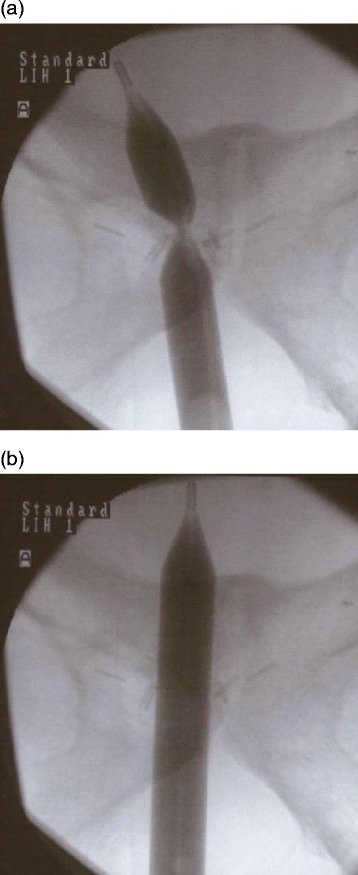
**a** Fluoroscopic image demonstrates the concentric deformity in the balloon at the site of the vesicourethral anastomotic stricture. **b** No deformity is noted in the balloon after dilation with high pressure inflation

After confirming the presence of VAS via endoscopy, a guide wire was inserted beyond the stricture under fluoroscopic guidance. The guide wire was inserted into the bladder before the dilation catheter was advanced over the guide wire into the bladder. The dilation catheter was positioned in the middle of the VAS, overlapping the normal urethra and likely including the external urethral sphincter. The balloon was inflated to 30 ATM pressure for 5 minutes, using pure contrast medium so that dilation of the VAS could be visualized (Fig. 2). Balloon dilation was performed three times with an interval period of 1 minute. After the procedure, a transurethral 16-Fr Foley catheter was placed until the following day.

Follow-up was performed via endoscopy. In patients with recurrent stricture, a second dilation procedure was performed. Recurrence of the stricture and the incidence of complications, particularly incontinence, were evaluated retrospectively.

## Results

The mean time from RP to the diagnosis of VAS was 9 months (range, 2–40 months) (Table 2), and eight patients (80 %) were diagnosed within 6 months after RP. Balloon dilation of VAS was technically successful in all patients, and no perioperative complications were recorded. All the patients were able to void spontaneously after the transurethral catheter was removed. Urinary continence before balloon dilation was controlled by only using a safety pad in all patients, but one patient complained about having to use more than one pads. The median follow-up after balloon dilation was 24 months (7–67 months). Eight patients (80 %) were successfully treated, without recurrence, following the first balloon dilation procedure. In the remaining 2 patients, recurrence of the stricture necessitated a repeat balloon dilation procedure, which was performed within 6 months of the initial dilation. No patient required > 2 balloon dilations.

**Table 2:** Time from radical prostatectomy to stricture and the outcomes following high pressure balloon dilation

Patient	VAS occurrence after RP (months)	No. of Stricture recurrences	Time to the first recurrence (months)	Follow up	Recurrence at the time of the last follow up	No. of pad (/day) pre dilation	No. of pads (/day) post dilation
1	6	1	5	67	-	1	5
2	6	1	3	53	-	0	0
3	3	-	-	31	-	0	0
4	3	-	-	31	-	0	0
5	3	-	-	27	-	1	1
6	4	-	-	20	-	1	1
7	40	-	-	16	-	1	1
8	4	-	-	14	-	1	1
9	2	-	-	22	-	0	0
10	22	-	-	7	-	0	0

## Discussion

Worldwide, open RP is performed as a standard procedure for organ-confined prostate cancer. VAS is one of the most common complications after open RP, although rates of VAS development vary widely [1, 2]. Occasionally, VAS can be associated with severe voiding dysfunction, and consequently a deterioration in quality of life. In both laparoscopic and robotic assisted laparoscopic RP, there are lower incidences of VAS, in the ranging from 0–3 % [8–10]. While there is no definitive cause of VAS, it has been suggested that the suturing technique is the most important preventative aspect [10].

Numerous approaches are available for the managing VAS, and much has been published on the various procedures (Table 3). Simple dilation using catheters or bougies is often performed as the initial treatment modality; however, this is associated with high recurrence rates. Cold knife incision is the most commonly performed invasive procedure, with high success rates [7, 11]. More recently, new modalities using bipolar electrocautery or the holmium YAG laser have been reported with acceptable efficacies [12, 13].

**Table 3:** Managing vesicourethral anastomotic strictures (VAS) following radical prostatectomy reported in the literature

	management	cases	Recurrence rate	Follow up (month)
Giannarini et al., [7]	Cold knife incision	43	26.0 %	12
Popken et al., [14]	Electricautery resection	15	53.3 %	12-72
Brodak et al., [12]	Bipolar resection	22	9.0 %	14-72
Lagerveld et al., [13]	Holmium YAG laser	10	0 %	3-29
Ramchandani et al., [6]	Conventional balloon dilation	27	41.0 %	1-84
Present study	High pressure balloon dilation	10	20.0 %	7-67

Transurethral balloon dilation is an established method of treatment for urethral stricture. The radial application of forces dilates the stricture, while avoiding the potentially traumatic shearing forces associated with sequential rigid dilation. Although transurethral balloon dilation has been performed for VAS previously, there are limited data regarding the outcomes in comparison to other modalities. Ramchandani et al. reported a recurrence rate of 41 %. In their series, the balloon of the dilation catheter did not expand completely [6].

We used a new urethral balloon catheter that achieves sufficient dilation against a more severe VAS using high pressure of up to 30 ATM. However, it is unclear whether a higher pressure of balloon dilation is more effective than conventional balloon dilation.

In the present study, balloon dilation was associated with a high success rate (80 %), and recurrent strictures could be controlled by performing repeated balloon dilation. However no patients required further treatment. Such findings are comparable to those associated with cold knife incision, which is considered the standard management for VAS.

Transurethral balloon dilation is simpler and less invasive than cold knife incision. Balloon dilation also has the advantage of a lower risk of urethral vascular injury. There is a wide range of complications associated with cold knife incision. Perineal hematoma and urethral hemorrhage are the most common complications ranging up to 20 % [15], and also de novo incontinence as a result of VAS has an incidence of 75 % [7].

However, no complications were reported in our study, except for worsened urinary incontinence in one patient (10 %).

Although our study was limited by the small number of patients and relatively short follow-up duration, dilation using this new high pressure balloon catheter appears to be an effective and minimally invasive treatment for VAS.

## Conclusion

Although balloon dilation for VAS has been performed previously, it was believed to have a low efficacy. We demonstrated excellent short-term results by using this new catheter, which can be inflated to a higher pressure than standard balloon catheters. High pressure balloon dilation appears to be a viable option for the managing VAS secondary to RP.

## Abbreviations

VAS: Vesicourethral anastomotic stricture
RP: Radical prostatectomy
ATM: Atmosphere
TURP: Transurethral resection of the prostate
EPE: Extra prostatic extension
RM: Resection margin
